# International Dental Federation

**Published:** 1902-02

**Authors:** 


### INTERNATIONAL DENTAL FEDERATION.

(Continued from page 54.)

### INTERNATIONAL COMMISSION OF EDUCATION.

*London, August 4, 1901.*

THE meeting was called to order at 10.30 A.M. by President Godon.

The following members of the Commission were present: Drs. Aguilar, Brophy, Cunningham, Godon, Hesse, Kirk, Roy, Patterson, Queudot, and Rosenthal.

According to the decision of the Executive Council, the delegates of the several national societies to the London meeting were admitted as adjunct members of the commission for the session of 1901. The members admitted under these conditions are: Drs. Haderup, of Copenhagen; Witthaus, of Rotterdam; Frank, Zsigmondy, and Weiser, of Vienna; Viau and Choquet, of Paris; Baruch, Quartermann, and Huet, of Brussels; and Bryan, of Basel.

Dr. Cunningham invited the members of the commission to visit the Institute of Technology.

The secretary-general read his report to the Commission of Education in the name of the Executive Council:

GENTLEMEN AND HONORED CONFRÈRES,—The International Commission of Education, which to-day holds its first meeting, has been created pursuant to the adoption of resolutions 14 and 16 presented to the International Dental Congress. [See *Dental Cosmos*, October, 1901, page 1163.]

Following the adoption of these resolutions the Executive Council of the International Dental Federation appointed the following members to the International Commission of Education: Drs. Aguilar, Brophy, Cunningham, Godon, Hesse, Kirk, Roy, Patterson, Queudot, and Rosenthal.

Besides the aforesaid members, the Executive Council having decided that all the members of the council were to take part in the labors of the International Commission of Education, the following names should be added: Drs. Harlan, Förberg, and Sauvez.

The Executive Council also decided to admit as adjunct members of the commission, with the right to take part in the discussions, the following delegates to the Executive Council: Drs. Haderup, of Copenhagen; Baruch, Quartermann, and Huet, of Belgium; Witthaus, of Rotterdam; Bryan, of Basel; Viau and Choquet, of Paris; Frank, Weiser, and Zsigmondy, of Vienna.

The number of active members of the Commission of Education can be increased by the Executive Council.

The commission will be represented by the following officers: A president, two vice-presidents, and a secretary.

The object of the International Commission of Education is as follows:

(1) To prepare a programme of the theoretical and practical subjects that the dentist should take up.

(2) To prepare reports for the following sessions on important questions of dental education.

(3) To present at the international dental congresses the questions of greater importance.

(4) To create and organize the necessary subcommittees corresponding to the several sections of the International Dental Congress of 1900.

(5) To create, maintain, and make closer the relations between the different dental schools.

(6) In a general way, to organize anything that may contribute towards the improvement of dental education.

The functions of the officers of the International Commission of Education are:

(1) To assure and to limit the work of this commission. (2) To bring together the several subcommittees. (3) To submit to the Executive Council the resolutions of the commission. (4) To examine the propositions which are addressed to it by the Executive Council or by the federations or societies.

Now that the constitution, the working, the *rôle,* and the purpose of the International Commission of Education have been pointed out, we will proceed to examine the conditions of dental education from an international point of view. It seems, however, impossible to enter into a limited discussion without beginning by a general one.

We must constantly bear in mind that the International Commission of Education appointed by the Executive Council must not try to impose the same system of dental education in all countries. The dentists of different countries form a series of dissimilar groups from the stand-points of number and professional status, and march in the road of progress with more or less advanced ideas according to the measure of maturity of the profession in every country.

We wish to insist upon this point in order to say once again that all the international commissions, of whatever nature, must be strictly careful not to interfere with such questions as are national, and to allow the latter to be freely discussed by the representatives of the particular countries concerned. Only in this way will it be possible for the commission to exist, and to work without awakening dissatisfaction. The different commissions should act as instruments of union, as organs of progress, and their members should, for the time being, entirely sink their personalities and nationalities in order to work only for an independent and high purpose. These commissions should limit themselves to discussing questions of a large and philosophical aspect, leaving aside the individual questions.

Dental education is at the present time the subject of much thought and consideration on the part of the leading members of the profession. Only yesterday Mr. Hutchinson, the newly elected president of the British

Dental Association, discussed this question. Some time ago Dr. Kirk discussed it in a very interesting publication in the *Dental Cosmos*. While we are here, a meeting consecrated to dental education is being held in the United States. We thus see that this question is discussed the world over.

The Executive Council has preferred to let the commission work freely on its own lines, without requiring from it any reports. What you have to discuss will really comprise the following points:

*First.* What preliminary knowledge is necessary for the dental student?

*Second.* What part of the medical and scientific subjects should be taken up, and at what time of the period of training should the study of them be pursued?

*Third.* What is the importance of theoretical technical knowledge?

*Fourth.* What is the importance of practical technical knowledge?

*Fifth.* What are the most appropriate names for the several titles now used all over the world?

We will call your attention to the reports of Drs. Godon and Roy to the Third International Dental Congress that we have brought once again before your consideration. Also to the reports by Professor Arkövy and Dr. Guillermin; to the seventh, eighth, and ninth resolutions adopted at the Congress, and to the recent article by Professor Kirk.

There cannot be the slightest doubt that with the foregoing basis, and with the officers that you will appoint, a meeting like this will produce the results expected from it.

You have all come from distant points with a disinterested purpose, in order to occupy yourselves with important educational questions which are of the noblest and most momentous character, as upon them rests the future of the profession.

We are certain that you will know how to elevate your discussions to the level of your exalted task, and that you will work with enthusiasm for the good of the profession and for the sake of humanity.

The secretary then read the following letter from Professor Arkövy:

To the Secretary of the Fédération Dentaire Internationale:

Dear Sir and Honored Confrère,—I highly regret that on account of poor health I find it impossible to attend the meetings of the International Commission of Education. In my capacity as a member of that body I shall send to Cambridge a report which I hope will in some way forward the desired purpose.

I beg you to accept the assurance of my best sentiments.

Prof. Arkövy.

The following report on Dental Education, by Professor Arkövy, was then read by the secretary:

### REPORT ON DENTAL EDUCATION.

Mr. President and Members of the International Commission of Education,—The Third International Dental Congress, in organizing the

International Dental Federation, had in view the establishment of dental education upon an international basis. Therefore I consider it necessary, before proceeding any further, to arrange a programme for the proceedings, and with the permission of the Commission I will take the liberty of proposing this *exposé* to serve as a foundation for further discussion, if the committee deems it fit for that purpose. Although no special directions were given by the Congress, or by the International Dental Federation to this body, there can be no doubt that the aims of the International Commission of Education are, first, to improve dental education, and secondly, to establish it upon an international basis. To the first proposition might be added, "if so desired," and to the second, "if possible."

These latter cautions show already the difficulties and obstacles most likely to be encountered in starting a question of this kind. Egotism in the national sense of the word, or rather patriotism, will make everybody inclined to consider the teaching system of his own country the best. As soon as we follow such a *manière de voir,* we will find no need for any amelioration at all, for the spirit of contempt regarding the educational systems of other countries implied in that feeling will prevent the adoption of a general basis for the teaching of dentistry. So the first inevitable step towards the advancement of this question is to discuss it from a standpoint of self-denial in the national sense, or by the adoption of a *sentiment of professional objectivity.* If we place ourselves upon such premises our *manière de voir* will be not only lucid, but also just and good for the interests of the profession. Before entering into the discussion of this subject I will ask the commission to discuss it from such a point of view.

Being well aware of the limited time at the disposal of the commission, as well as of the many questions that it will have to consider, I will try to be as short as possible and will enter into the subject *in medias res.*

The teaching systems, although numerous and considerably different in most of the countries, may be divided into three kinds: 1. The school system. 2. The university system. 3. The mixed or school and university system. In the first case the school is the teaching body and in several cases also the examining body. In some only a special surgical body confers the dental diploma (England, Australia, America). In the second case a university is the teaching body, and no special diploma is issued (Austria-Hungary). In the third case either a school or a university is the teaching body and the state boards confer the special diploma (Russia, Germany, State of New York, Holland, Denmark, and Sweden). There is not the slightest doubt that each system has its pros and cons. There is no need to enter here into a discussion of their respective values, as they are beyond the reach of an international commission, and we may fairly acknowledge that either of the systems mentioned, if properly conducted, may serve the purpose for which it is intended,—*i.e.,* the formation of good dentists. If we review the subjects that are taught in the several systems of education alluded to, we will soon find out what the differences in this direction are.

Dr. Kabo took the trouble of collecting the data for the synoptic table that I am joining to this report.[1] The plus signs denote the subjects that are taken up in the courses, and the minus signs those that are not. Before reviewing in a specialized manner this synoptic table, we will have to consider in the first instance the components of the present educational system, which may be divided into: 1. The preliminary education. 2. The professional education. Each of these questions contains a series of subdivisions regarding scientific and practical requirements.

Already preliminary education seems to be one of the most divergent phases of this question, because the so-called middle school system is so different in every country. For instance, in one the preliminary education is most elementary, while in others a bachelor's degree in arts, or its equivalent, constitutes the entrance requirement. It cannot be doubted that a good and a fuller preliminary education must necessarily elevate the standard of professional education. As there are no international means at our disposal by which to bring about uniformity in the problem of preliminary education, the only thing that we can do is to point out the defects of a low standard of preliminary education. Everybody will agree with me, that it is highly desirable that every dentist should be first of all a properly educated man, and after that a good professional man; that a properly educated man will make a better professional man than an improperly and defectively educated one. Therefore if any propositions are to be made for the improvement of professional education they should effect professional education at first in order to raise the requirements to the point where they stand to-day in other countries,—*i.e.*, baccalaureate degree and gymnasium certificate. We must insist again on the desirability of raising the standard of preliminary education, if not suddenly at least by degrees, in order to suit it to a high standard of dental education.

Professional education is subdivided into (1) theoretical and theoretico-laboratory parts; (2) into a practical part connected with theoretico-practical courses. (The latter may be more properly called by adjectives "clinical" and "operative.")

There are several important questions connected with professional education, the most important being whether all the branches including medical subjects should be taught in the professional schools? whether that is the better system, or if it is more advantageous that the medical subjects should be taught in the medical schools. Dr. Ch. Godon, of Paris, in a recent publication and in accordance with Dr. Roy's opinion, expresses himself thus: "It is advantageous that the complete education should be given in the dental school, where science and medicine can be better adapted to the needs of dentistry."

In my opinion there are strong arguments against this suggestion. First of all, it cannot be denied that medical schools are more competent to teach medical subjects than other schools could ever be; secondly, if only certain parts of medicine are picked out and adapted to the study of

---

[1] On account of lack of space to give to this extensive table, the publication of it is here omitted.

dentistry, it would mean, if we are permitted to make the comparison, the teaching of a language by teaching by heart some sentences such as "Good-morning," "Good-night," etc. Any person that would attempt to learn a language thus, I think, would never acquire it. The plan as proposed by Drs. Godon and Roy should be abandoned.

The second important question is whether the medical studies shall be taught before, after, or simultaneously with the theoretical ones.

Dr. Godon is of opinion that a parallel teaching is preferable to any other. I am sorry I again cannot agree with Dr. Godon. The best argument for supporting a contrary view is the example of the several old universities and medical schools, where parallel studies of theory and practice do not exist. If a student would learn anatomy, materia medica, or pathological anatomy at the same time that he does surgery, he would soon be confused, not being able to fully comprehend either of them. In his despair he would study superficially, and could thus never become a thorough physician or surgeon. On the other hand, if he had previously gathered sufficient theoretical knowledge, he would soon find out the practical application of his knowledge. If the students should forget some of the knowledge previously acquired they could easily gain it again, keeping pace in that way with the practical teaching.

The third important question was raised by Dr. Godon when he pointed out the superiority of the teaching of the École Dentaire of Paris by bringing forward what he calls a most favorable proportion between the time spent on theoretical studies and that spent on practical subjects. In his statistics he shows a total of 4212 hours, out of which 980 are spent in theory, while 3266 are spent on practical subjects. By these figures we can see that the proportion between the time devoted to theoretical work and that devoted to practical work is as 7.39 is to 30.39. Even Switzerland, with its proportion of 21.44 devoted to theory and 17.44 to practice, was put in the shadow by the Paris proportions.

I am again sorry that I cannot agree with Dr. Godon. In the first place I cannot see the utility of such a compilation of figures in the solving of the problem of dental education. Does it mean that the less theoretical and more practical knowledge a dental programme contains the better it is? The astounding effect that such a statement produced upon me induced me to collect similar statistics on the medical studies of a European university. I took the University of Budapest as an instance. The table already referred to and annexed to this report shows curious figures compared with the above statement. The total medical instruction is given in five years (ten semesters), or 8632 hours, out of which 5002 are devoted to the study of theoretical subjects while only 3630 are devoted to practice. This proportion, with very little difference, is common in almost all the old universities and medical schools. Now, would anybody say that medical men are not up to their profession or that medical education, so many hundreds of years old, has always been bad because the medical man is taught more theory than practice. It will be noticed also that in any branch of scientific knowledge more theory is studied than would be necessary in the practising of it. Please do not confound scientific professions with handicrafts. If you turn your figures in favor of practice, then a handicraft man will result.

The professional education proper, as can be seen in the synoptic tables, offers just as many differences as the various points of the educational problem. The main object in that table is to demonstrate that the theoretical subjects, such as anatomy, physiology, general pathology, chemistry, physics, and materia medica, are only taught in their applied relations to dentistry. Only in a few schools are they taught in full (Germany, Switzerland, Sweden, Austria, and Hungary), and even in those schools the subjects alluded to do not form part of the programme of examinations; as a consequence the students neglect to study them. Other subjects of the theoretical department, such as bacteriology and pathological anatomy, although of very great importance, are in certain schools completely omitted.

The practical subjects are, as a rule, much better taught in the school and mixed systems. By the university system the medical education is complete, as the candidate must be a medical graduate.

The defects in the teaching of the practical subjects may be pointed out as follows: First of all, it seems astonishing how a proper education in a medical specialty—and dentistry is such a specialty—may be imparted to students without a thorough teaching of general and operative surgery. How can a dental student comprehend clinical pathology—the main subject of his own specialty—without knowing the details of wound-healing, etc., details that are only supplied by general pathology? On the other hand, how can students of dentistry, who, even after receiving as they do in some schools instruction in oral surgery, know nothing at all about it, treat alveolar diseases, even if they were only consecutive appearances of strictly dental disorders? Medicine—the mother science—forms part of the dental curriculum only in England and in France. Austria and Hungary need not be mentioned, as there the M.D. degree is required from all those that desire to take up the study of specialties.

I do not see why a dental practitioner should be ignorant of the nature of such disorders as fainting, hystero-epilepsy, etc. But we do not want to go so far as that. On glancing at the synoptic table one will readily see with how many internal diseases the dentist should be familiar in order to fill teeth on a scientific basis and not on empirical lines.

In enumerating all these drawbacks to dental education as carried on all over the world, I have been obliged to confine myself strictly to the pointing out of all these errors, not being able, on account of limited time, to discuss the plans and ideas that should follow from these remarks and that would remedy the defects alluded to.

### RÉSUMÉ.

In summing up the data and suggestions brought forward in this address, we have to keep to the distinctions made hitherto.

I. The teaching systems, different as they may be in different countries, do their service equally or nearly equally well for the professional education as soon as they are properly conducted, and aiming to a higher or the highest standard possible to attain. So the principal point of dental education does not lie in the system adopted. (College, mixed, university system.)

II. The preliminary education has to be in accordance with the quantity and quality of knowledge to be gathered by the student later on,—viz., in subsequent years. Such being the case, he ought to be equipped with as much preliminary knowledge as may be adequate to the task in view. It has been stated, and by several schools approved, that the bachelorship in arts, or the certificate of maturity, are the best preliminary requirements for further study in the line.

III. For teaching medical subjects, there can be no doubt about the superior competency of universities and medical schools, while the competency of dental schools as institutions for an applied teaching is to be considered as far beyond doubt. In this way we are obliged to contemplate for dental education not a single but a double teaching in the subjects under consideration.

IV. The time when the theoretical teaching should take place—as has been demonstrated above—seems to be best arranged when the example of medical schools is followed,—viz., theoretical subjects precede practical teaching.

V. Proportion between the time spent in theoretical studies as opposed to practical ones. If there should be any such definitely established, a considerable disproportion between the two ought to be arranged, taking into account that a much larger amount of theory is required to a thorough practice in a scientific profession.

VI. If any improvements were intended to be made upon the present dental education—and there is a sensible demand for it—they should be directed towards subjects of teaching hitherto partly or entirely neglected, or insufficiently cultivated; as such may be considered, pathological anatomy, bacteriology, internal diseases, anatomy, physiology, histology, general pathology, chemistry, physics and materia medica, general surgery, and surgery in general. How much time, or how much *more* time, should be allotted to each of these subjects is a matter of private concern for the individual schools and teaching bodies.

VII. The preceding point has already indicated the necessity of extending the duration of the curriculum. So many more subjects and so many more hours of lectures as would be required in adopting the above improvements would involve, in an approximate estimation, the extending of the curriculum for one year (two semesters).

VIII. In connection with that point, and in order to meet the exigencies of practice, it would be advisable to establish an extra six months' hospital or private training, *after passing* the professional examination, approbation, or whatever constitutes the finishing of studies. Such an arrangement is necessary and is already in vogue at some European universities for medical practice, amounting to a full year's hospital or clinical training.[1]

---

[1] I wish to be understood that training is not to be confused with teaching in practical subjects, the former being subsequent to the latter

IX. As a general observation it must be mentioned that odontotechnical lectures—theory and practice, including especial training—require more attention and time in schools of the mixed system, and still more in the pure university system. The idea of a separate training-school in dental mechanics, as suggested by Mr. G. Cunningham, Cambridge, England, is a good one, as it would afford such an opportunity to dental students as hospitals and university clinics give to the medical student, or rather young practitioner not yet in independent contact with the public. But the guidance in such a place ought to be a perfect one.

X. The requirements, if raised to such an extent, are bringing the dental student so close to the medical that there is only a further step necessary to turn him into a medical man. We cannot conceal that there is a strong tendency, at least in Europe, and also some half-suppressed groans were audible from the " other side of the water," for the attainment of a higher professional qualification, even of a university degree.[1] Slowly, but I believe surely, the profession will arrive one day at a point where there will be no difference between the dentist, as a professional man, and the medical man.

We were delegated and are assembled to do our best and to confer as much as we are able to the advancement of the interests of the dental profession. There is not one item among them of such high importance as is the professional education of succeeding generations. There can be no nobler task and no more sublime mission than the one bestowed upon us. As soon as we acquire information about the conditions existing in that educational line we could not, and I am sure we would not, restrict the frank and candid pronouncing of our conviction, disregarding whether this would affect feelings or not. We have seen there is a good deal to be done in that way; if you agree with my disclosures as demonstrated in this address I am convinced you will do a great service to the profession. We have no power, no jurisdiction at our disposal, but nobody will doubt that there is an overwhelming power in a pronouncement of a committee invested with an international character.

If your decisions should have no more effect than that of giving indications as to how dental education should be carried on, and what development and evolution it should undergo in future, then you have performed your noble task in a worthy way.

---

and differently conducted in its realization. This kind of teaching is supplied by lectures and demonstrations and can be imparted to a large audience; while the training is carried on, under guidance, at several places (hospitals, clinics, private practice), and the pupils can only be grouped in these cases in small numbers,—otherwise a training would be a failure, and to the detriment of the students.

[1] I fully agree with the views of Mr. Thomas Geddes, M.D., as developed in " A Plea for the Specialty of Dentistry and for University Recognition." Journal of the British Dental Association, July, 1901.

After the reading of Professor Arkövy's report the following communication by Dr. L. Guillermin, the Swiss delegate to the International Dental Federation, was read by the secretary:

### DR. GUILLERMIN'S COMMUNICATION.

Considering the difficulties met with in the liberal professions one is inclined to question whether the governments and professional societies are following a wise policy by facilitating in an extra liberal manner the access to the different professions. The attention of those bodies should rather be directed to the improvement of methods and to the selection of candidates.

The difficulties alluded to are most detrimental from a professional as well as from a social stand-point. In the professions of law and medicine there are men who should be at the top of the ladder, as far as science, honor, and dignity are concerned; but instead, we see them mixed up in the worst kinds of compromises, becoming the cause of scandals whose effects are felt by the entire profession. As the result of practices of this kind the public suffers and the practitioner becomes completely ruined. We are rapidly reaching a very plethoric state in dentistry, and for the general interest, as well as for the individual interest of the young student, one should not encourage him unless his qualities fit him for the profession that he wants to follow. It devolves on the parents, professors, and educators to make the selection while there is time to prevent the public from doing it when it is too late.

At the present time success does not always crown the efforts of the most deserving graduates, because they are lacking in one or more of the numerous qualities required for the practice of our art. How many lamentable confidences have I not received during the last twelve years of the thirty-two of my professional life.

How should a candidate be recognized as being fitted for the practice of dentisry? Manual skill and well-developed intellectual faculties seem to me to be indispensable qualities for the formation of a good dentist. Dexterity and intelligence constitute very high requirements, but nevertheless, they only represent the minimum that could be demanded.

In connection with this idea I must say that I admire the creation of the Institute of Dental Technology by Dr. George Cunningham, as there it can be easily ascertained what the disposition of the student is, and he himself can find it out, as no efforts are made to conceal the truth from him. We have only considered the natural gifts of the individual; their presence in a given person will assure the successful completion of the important and difficult studies leading to a diploma.

An idea which is making its way into Switzerland is that in the near future the degree of doctor of medicine will be required for the practice of dentistry, this is already the case in Italy and in Austria; in France one of the most illustrious representatives of dental science, Dr. Godon, has recognized this necessity by taking his degree in medicine. In this country we see quite a number of dentists, holders of the medical degree, enlightening with their intelligent activity the most obscure problems of our art. But in any case (doctor of medicine or *médecin dentiste*) complete secondary studies are required. These comprise at least a knowledge of Latin (*maturité reale suisse*) if not that of Latin and Greek (*maturité classique*). The obtaining of this diploma, which corresponds to the French baccalaureate degree, is in the majority of cases a proof of normal intellectual faculties. After this a year is spent in scientific studies (physics, chemistry, botany, and zoölogy), and if finished successfully it confirms the capacity of the candidate. These are the preliminary requirements in Switzerland, and only when completed do the medical studies properly speaking begin. It remains to ascertain the manual skill of the candidate. For this purpose he could be made to spend two hours daily in prosthetic work in the laboratory of a dentist or in a dental school in conjunction with his medical studies. At the end of this year an examination would decide the ability of the student, and if his manual skill be not clearly demonstrated, he could only be a physician. It should be demonstrated that in order to make a good dentist, it takes, besides the necessary intelligence, more time and dexterity than to make a good physician. I say physician, not surgeon. Once at this point, the student would devote a few hours every week to dentistry. After obtaining the medical degree he would take up dentistry exclusively during four semesters, and could then be considered as being a dentist. It is noticeable that the majority of physicians who want to become successful practitioners spend two years as internes in the hospitals. These two years would be represented by the dental interneship.

Dentistry, or stomatology as you want to call it, would then become an integral part of medicine, a specialty, just as ophthalmology, laryngology, gynæcology. In fact, is there any medical specialty in which the practitioner is in closer daily relations with the general state of the patient than in dentistry? Is it not necessary to have a large medical experience in order to administer general anæsthetics? Is it not sometimes necessary to constitute a general treatment previous to the buccal operations, and is not one sure of failures if dental operations are performed upon anæmics, rachitics, malarials, syphilitics, diabetics, or neurasthenics without the previous administration of iodides, phosphates, quinine, etc.?

The analysis of normal and pathological saliva, the diagnosis of neoplasms, auscultation, the necessary authority in order to obtain from the patients information as to the period of pregnancy, the catamenial state,—do not all these conditions belong essentially to the province of the medical art? It would be necessary to review the entire nosography, all the physiological states, in order to fully comprehend the constant relationship between the oral cavity and the organism. There is no doubt that the dentist acquires the knowledge and necessary authority, but only after years of practice and hard and difficult supplementary study.

We are persuaded that this solution, the obtaining of the degree of doctor of medicine, is the only means by which dentistry can reach that dignified position, that social elevation, that it partially possesses through the efforts and sacrifices of Godon, Dubois, Brasseur, and of others, and

I am mentioning only the French ones. Besides, the candidates for the diploma of *dentiste médecin* would not remain any longer satisfied with the teaching of the professors; they would then be in a position to do original work. What would become of the dental schools? They would rid themselves of the present scientific department, which I must say is wonderfully organized.

On account of the growing importance to which this will attain, the schools will be transformed into true schools of medicine, with their professors, their dissecting-rooms, their laboratories, and their hospitals. The struggle with the official schools will not be possible any more, nor the students be called upon to attend the lectures therein given. It seems to us that the dental schools should become especially *dental*, and should lose the character of schools of stomatology that they possess at present. They should teach exclusively prosthesis and operative dentistry, which in themselves constitute a wide field. This transformation will not take place at first, but we believe it to be unavoidable, and either the new dental schools or the old ones should force it. In the mean while we believe that nine to ten semesters of study should be required, provided that this measure is adopted by all the schools. The ideal would be that a complete dental school, an autonomous one, with its professors and its own organization, should be annexed to two or three of the principal hospitals in the great cities, and to one in the smaller ones. They would be the analogue of dispensaries devoted to other medical specialties.

Regarding the international federation of dental schools, I must say that the majority of professors of our Swiss schools reserve their opinion by adopting at present this measure which can only be consequent to the adoption of a uniform programme; they fear to make the schools lose their individuality. The differences in method, the predominance or equality of the theoretical and technical elements, are dictated by usage. The general ideas, the aspirations of the different countries, produce a diversity that tends to bring about a healthy emulation between the schools and causes the different institutions to pursue special studies, and this to the benefit of the profession.

I will add to these considerations, which reflect in a general way the ideas admitted in our teaching spheres, as well as my personal convictions, copies of two reports, one from Professor Billeter, professor at the Faculty of Medicine of Zurich, and also at the dental school of this city, and the other from Professor Métral, Médecin Dentiste, professor at the École Dentaire de Genève.

Please present to my honorable colleagues of the International Dental Federation my regrets for my inability to be present at their meeting.

### REPORT BY PROFESSOR BILLETER.

Dentistry is a specialty of medicine, and hence the preliminary education for the stomatological candidate in Switzerland is as follows:

1. A *gymnasium* education which is equivalent to the upper classes of the French *lycée* and altogether similar to that of the dentist.

2. A *propendetique* education, similar to that of the physician.

3. Now, as far as the professional education properly speaking is concerned, the same attention is paid to the technical as to the operative teaching. As our art has made considerable progress in metallurgy, ceramics, and surgical prosthesis, it will be necessary that the curriculum should embrace all these specialties. As a consequence of these facts, professional education will require in Zurich four semesters instead of three, as is the case at the present time.

It is necessary that in all countries where dental schools exist at present all these specialties should be taught. But, as far as the details of the teachings are concerned the liberty of arranging them should rest on the individual countries.

REPORT BY PROFESSOR MÉTRAL.

I believe in bringing about as far as it is possible uniformity in all the dental schools.

The time devoted to the study of dentistry should be the same as for medecine,—*i.e.*, nine semesters, four to be devoted essentially to theoretical studies, general education, national sciences, medicine and surgery, and five semesters to be devoted altogether to the practical subjects and to the theoretical studies which concern them directly.

A course in bacteriology would be introduced into the theoretical part of the curriculum and the students would be required to take a course in auscultation. The practical side of dentistry would develop advantageously through specialization of the subjects of anæsthesia and orthodontia.—*Dental Cosmos.*

(To be continued.)

